# Antioxidants in Extra Virgin Olive Oil and Table Olives: Connections between Agriculture and Processing for Health Choices

**DOI:** 10.3390/antiox9010041

**Published:** 2020-01-02

**Authors:** Barbara Lanza, Paolino Ninfali

**Affiliations:** 1 Council for Agricultural Research and Economics (CREA), Research Centre for Engineering and Agro-Food Processing (CREA-IT), Via Nazionale 38, I-65012 Cepagatti (PE), Italy; 2 Department of Biomolecular Sciences, University of Urbino “Carlo Bo”, 61029 Urbino (PU), Italy; paolino.ninfali@uniurb.it

**Keywords:** extra virgin olive oil, table olives, phenols, tocopherols, secoiridoids

## Abstract

This review focuses on the conditions required to increase and maintain the antioxidant nutrients in both extra virgin olive oil (EVOO) and table olives (TOs) from the agronomic and technological practices to the gastronomy. The main antioxidants of TOs and EVOO are phenol alcohols and acids, secoiridoids, lignans and flavones, all of which possess the ability to prolong the oil’s shelf-life and exhibit healthy properties for humans. The precise detection of secoiridoid derivatives remains the breakthrough for the nutritional and health quality certification of extra virgin olive oils (EVOOs) required for EFSA health claims. To attain the necessary antioxidant quality in both EVOO and TOs, it is necessary to hard focus on the several steps in the production chain, including olive cultivar, agronomic conditions, harvesting methods, and transformation technology. The quality level is maintained if the storage conditions aim to minimize the oxidative processes that occur due to oxygen and light. In terms of minor polar biophenols, there is disagreement on which between the organic or conventional EVOOs show higher concentration values. The strict disciplinary of production of protected designation EVOOs does not ensure higher phenol values in comparison to the artisanal EVOOs. In gastronomy, the EVOOs are preferable to seed oils, particularly during frying vegetable. The EVOOs show higher heat stability, linked both to the fatty acid composition and the phenol content, that is important for preventing fatty acids oxidation. Concerning TOs, the commercial presentation includes olives and olive paste. Both products show a remarkable loss of natural antioxidants after pasteurization and during storage as the thermal treatment mostly impacts on TOs secoiridoids.

## 1. Introduction

Extra virgin olive oil (EVOO) and table olives (TOs) are central foods in the Mediterranean diet [1,2]. Scientific advances on their health benefits suggest to increase their use to prevent chronic diseases [3] and marketing strategies are searched for orienting the choice towards quality products [4]. The lipidomic science provides the most convincing evidence for the need to carefully choose the alimentary fats. The lipidome analysis may reveal which lipids are used in the diet from the detection of the lipids enclosed in the lipid bi-layer of the erythrocyte membranes [5]. Those having a high dietary intake of animal fats or hydrogenated seed oils shows a lipid pattern significantly different from those consuming EVOO or TOs [6]. This different lipid composition may have an impact on the function and permeability of the cell membrane [5].

Italy produces 15% of the world EVOO market and it is the first EVOO world consumer. From the most recent population surveys, it emerges that Italian consumers are attracted to the increase EVOO quality as they are appreciating the health benefits of EVOO (ISMEA 2019) and can recognize the organoleptic properties, associated with agronomic and technological factors (ISMEA 2019). Information on the benefits of EVOO in gastronomy, is available in the literature, on the web, as well as in the media. The ability to choose high-quality EVOO and TOs and their correct use in gastronomy, i.e., how the products are used for domestic cooking, are two inseparable aspects to support the efficacy of the two products in health protection. The strategies to spread scientific and technological knowledge to construct and increase people awareness of quality choices are under the attention of research groups in other countries, particularly in Spain [7,8].

The high quality EVOO is considered as a true pharm-food. This property is due both to the fat composition, i.e., high oleic acid concentration, which ranges from 56% to 84%; the essential polyunsaturated fatty acids: linoleic acid ranging from 3.5% to 21% and linolenic acid <1.5%. Besides, EVOOs contain a relevant concentration of efficient chemopreventive molecules, including tocopherols (vitamin E), β-carotene, and phenolic compounds (PCs).

**Figure 1:**
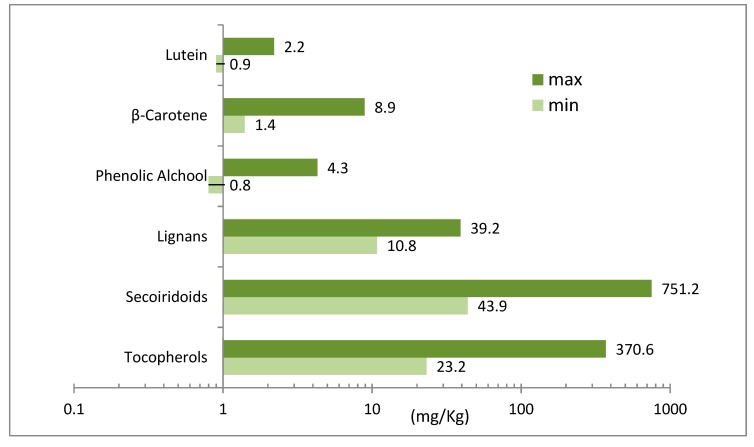
Minimum and maximum concentration of the main antioxidants in extra virgin olive oil (EVOO) from different Italian regions. Values are compared using a logarithmic scale. Adapted from Antonini et al. [10], Ninfali et al. [11] and Psomiadou et al. [12].

The PCs are grouped under five categories: a) the phenolic acids; b) the phenolic alcohols, such as the 3,4-(dihydroxyphenyl)ethanol (3,4-DHPEA or hydroxytyrosol) and the *p*-(hydroxyphenyl)ethanol (*p*-HPEA or tyrosol); c) the secoiridoids, such as the dialdehydic form of decarboxymethyl elenolic acid linked to hydroxytyrosol (3,4-DHPEA-EDA), called oleacein, and the dialdehydic form of decarboxymethyl elenolic acid linked to tyrosol (*p*-HPEA-EDA), called oleocanthal, the 3,4-(dihydroxyphenyl)ethanol elenolic acid (3,4-DHPEA-EA), called isomer of oleuropein aglycon, and the *p*-(hydroxyphenyl)ethanol elenolic acid (*p*-HPEA-EA) or ligstroside aglycon; d) the lignans, such as (+)-1-acetoxypinoresinol and (+)-pinoresinol; e) the flavones, like apigenin and luteolin [9,10]. Figure 1 shows the minimum and maximum concentrations of antioxidants of EVOOs. Data show that among the PCs the highest concentration is provided by the secoiridoids, as compared to the other antioxidant compounds.

**Figure 2:**
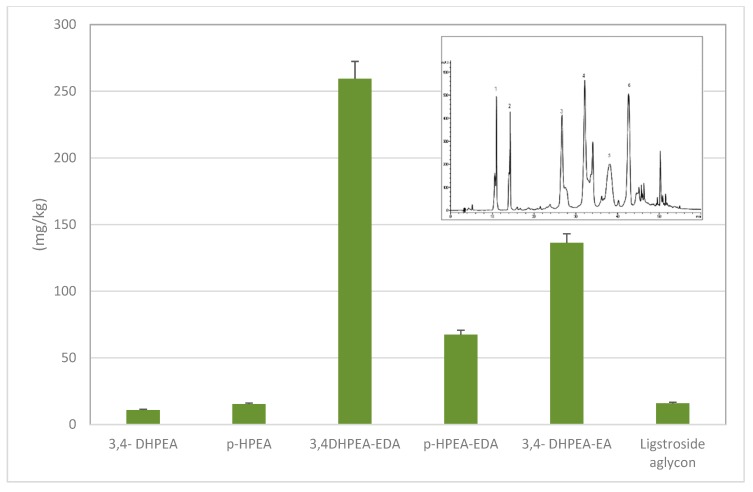
Concentrations of individual phenolic alcohols and secoiridoids in a typical EVOO, measured by HPLC-DAD analysis. The inset shows the chromatographic profile with the numbers on the top of the peaks indicating respectively: 3,4-DHPEA (1); p-HPEA (2); 3,4-DHPEA-EDA (3); p-HPEA-EDA (4); 3,4-DHPEA-EA (5); ligstroside aglycon (6). The mobile phase was water and 0.2% acetic acid (solvent A) and methanol (solvent B); the flux was 1 mL/min. The elution gradient was made as follows: 95% A/5% B for 2 min, 75% A/25% B for 8 min, 60% A/40% B for 10 min, 50% A/50% B for 16 min, 0% A/100% B for 14 min; the latter gradient was kept constant for 10 min, to return at the initial phase. Adapted from Selvaggini et al. [9] and Antonini et al. [10].

Figure 2 shows the relative concentrations of the individual phenolic alcohols and secoiridoids in EVOO. The concentration of 3,4-DHPEA-EDA clearly overcomes the others.

The secoiridoids are produced in the secondary metabolism of the terpenes in the drupe pulp, but they are also present in the pericarp and the stones [13,14]. The secoiridoids of EVOO are mainly derived from oleuropein, demethyloleuropein, ligstroside, using enzymatic hydrolysis, carried out by the beta-glycosidase, of the olive fruit [15]. The extent of their concentrations depends on the cultivar, maturation stage and methods of harvest and processing [11,16]. The oleuropein increases during the fruit maturation and it may be considered as an indicator of the phenolic maturation of the olives. It is worth noting that there is a significant difference between the time of phenolic maturation and the time of industrial maturation, i.e., the period where the oil yield reaches the maximum [17]. The 3,4-DHPEA-EDA, derived from oleuropein, is responsible for the bitter taste, whereas *p*-HPEA-EDA, derived from ligstroside, is responsible for the pungent taste [17]. Bitter and pungent are therefore positive characteristics of the EVOO as linked to health benefits, such as most of the PCs of the EVOO [18]. Biological activities of the secoiridoids consist principally in: depletion of oxidized low density lipoprotein; increase of the plasmatic antioxidant capacity; protection from inflammatory reactions [19,20,21]. Concerning the latter aspect, the oleocanthal was shown to be molecularly active within the cell, in a very similar way as ibuprofen [22]. Moreover, the 3,4-DHPEA can reduce the effects of two pro-inflammatory cytokines: TNF-alpha and Interleukin 1 B (IL-1 B) [23]. For a wide description of the many health benefits, excellent reviews have been published [23,24].

The beneficial properties of EVOO phenolic compounds may be potentially provided to a greater extent by TOs, due to their very high content of secoiridoids in comparison with EVOO. Table olives show phenolic amounts ranging between 100 and 400 mg/100g of edible portion (e.p.) of olive fruit, represented by the flesh (epicarp and mesocarp without endocarp), which roughly corresponds to the same quantity provided by 1 kg of extra virgin olive oil [25]. The main phenolic compounds found in table olives are: simple phenols (phenolic alcohols and acids); secoiridoids such as oleuropein, demethyloleuropein, ligstroside, and derivatives such as 3,4-DHPEA-EDA and p-HPEA-EDA; hydroxycinnamic acid derivatives as verbascoside and iso-verbascoside; lignans; flavonoids.

This review focuses on the EVOO and TOs nutritional quality and the conditions able to increase and maintain the antioxidant nutrients, with particular attention to agronomic and technological practices as well as to the gastronomy. Analytical methods, adopted to provide the concentrations of the antioxidants are discussed as well.

## 2. Analytical Problems in the Phenol Evaluation

The precise detection of EVOO hydroxytyrosol and its derivatives remains the breakthrough for nutritional and health quality certification [26,27]. The European Food Safety Authority (EFSA) stated the admissibility of the health claim for EVOO at two conditions: first, the olive oil must contain at least 5 mg of hydroxytyrosol and its derivatives (e.g. the oleuropein complex and tyrosol) in 20 g and secondly, the claim must be matched with indication that health benefits may be obtained by consuming 20 g of oil per day (Regulation EU N. 432/2012) [28].

The simplest method to evaluate the whole concentration of al phenolic compounds in EVOO is given by the official colorimetric method, based on the Folin–Ciocalteu reagent, which permits to evaluate the reducing capacity of all phenols [29]. The method is not specific and the results differ from those obtained by the High-Performance Liquid Chromatography (HPLC) methods [27].

The International Olive Council (IOC) during 2017 [30] has proposed a method based on the HPLC technique. A UV detector at 280 nm or a diode array detector (DAD) is needed; siringic acid is used, as internal standard and tyrosol, as external standard, for the calibration curve. Data are expressed in mg/kg of tyrosol.

Many other methods have been proposed in the EU countries, to overcame some problems unresolved by the proposed IOC analytical method. It has been observed that if quantified referring to tyrosol, oleacein, and oleocanthal, cannot be correctly estimated due to the different UV response factor and different molecular weights [31].

A method based on the calibration curves obtained with oleacein and oleocanthal, which now exist as commercial standards, would be able to provide reliable detections. The application of DAD and fluorescence detectors, were proposed to accurately evaluate the secoiridoids and the lignans, respectively [9].

Other scientists proposed to detect secoiridoids, after the acid hydrolysis in the oil itself, in such a way to have all of the secoiridoids, represented in the HPLC chromatogram by two well-defined peaks: one for p-HPEA and the other for 3,4-DHPEA [31,32]. Other research groups proposed to carry out the secoiridoids hydrolysis in the ethanolic extract [26] and other groups suggested to perform the hydrolysis followed by the hydroxytyrosol and tyrosol derivatization for the gas chromatographic analysis [33].

In many EU countries, it emerges the need to make the analysis of EVOO secoiridoids derivatives by means of a sole and simple method, easily accessible to labs, in order to support the olive oil producers in their territories.

In this light, a strict debate is going on inside the IOC analytical expertise panel to find one official method for detection of hydroxytyrosol and its derivatives in EVOOs, to be proposed for assigning the EFSA health claim.

The analysis of the tocopherols, is another important issue in the quality detection of EVOO [12]. The tocopherols contribute to the oil stability during the storage and, in synergy with the phenol compounds, develop an important role in the protection of the living cell membrane and reduction of the oxidative reactions on the lipoproteins [23]. In EVOO the analysis regards principally the α -tocopherol, because of the negligible amounts of the β- and γ –tocopherols [34].

This analysis is performed with the oil diluted with hexane and then injected into the HPLC equipment, working in the direct phase separation method; the HPLC may be endowed either by UV-Vis or DAD detectors [34]. A fluorescence detector can also be profitably used [12].

**Figure 3:**
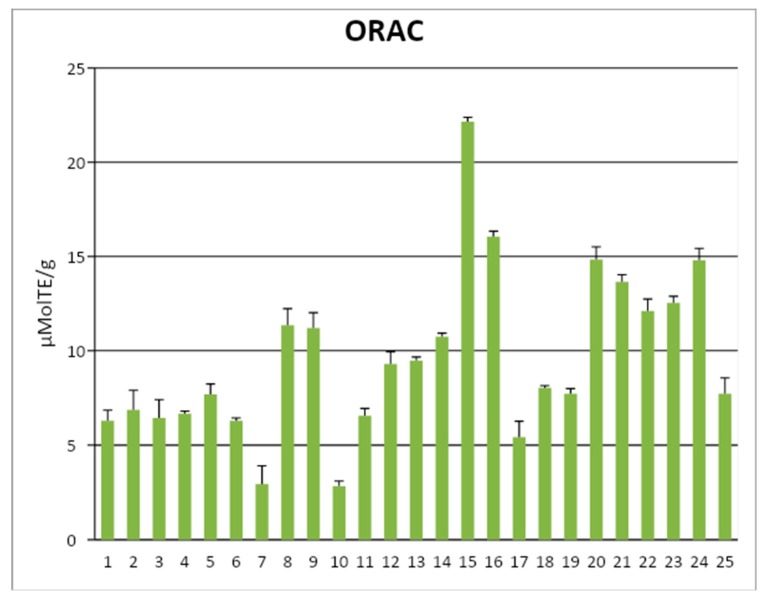
Heterogeneity of EVOO antioxidant capacity measured with the ORAC method on 25 oil samples. Values allow us to distinguish four categories of antioxidant capacity, putatively assigned by us in the following ranges: 1–4, low quality; 4–8, intermediate; 8–12, high; >12, top quality. Adapted from Antonini et al. [27].

As far as the detection of the antioxidant capacity of the EVOO, we measured this capacity with the oxygen radical absorbance capacity (ORAC) method, which quotes the efficiency of the antioxidants to reduce the peroxyl radicals generated in the reaction mixture, in comparison with an analogue of the vitamin E, called Trolox [11]. By the ORAC method, we ranked several EVOO in micromoles of Trolox equivalents/g (i.e., ORAC units) and we suggested four categories of ORAC quality. The ranges were the following: 1–4, low-quality EVOO; 4–8, intermediate; 8–12, high; >12, top-quality [27]. Figure 3 shows an example of the ORAC value obtained on 25 Italian EVOOs analyzed in a seasonal production [27]. The ORAC values strictly correlated with the phenolic content [11].

Other methods, such as 2,2-Diphenyl-1-picrylhydrazyl (DPPH) assay, ABTS+ or lipid peroxidation inhibition have been profitably used [35,36,37]. In parallel with the antioxidant capacity, which is evaluated on hydrophylic phenols from the EVOO ethanolic extract, it is useful to evaluate the amount of products derived from the lipid peroxidation in the oil itself. The lipid peroxidation effect may be provided by merchandise parameters, such as peroxide index, UV extinction coefficients calculated from absorption at 270 and 232 nm. The detection of the dialdehydes, such as hexenal, hydroxynonenal, and malondialdehyde, by reverse phase HPLC method has been also profitably used [38].

## 3. Antioxidants in EVOO Depending on the Extractive Technology

**Figure 4:**
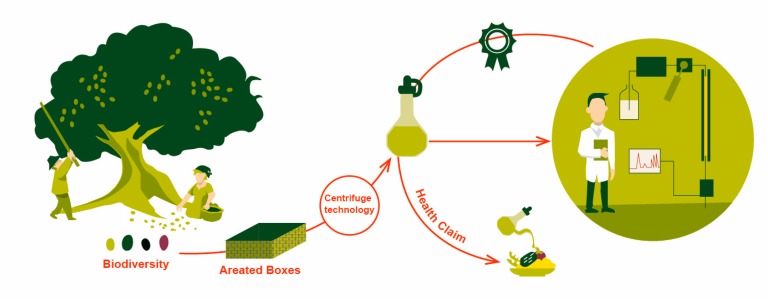
Graphical abstract of the steps for the oil quality management concluding with the connection to territorial labs for certification by an official analytical method.

The Italian olive producers are distributed on 820,000 farms, with 5000 oil mills available (ISMEA 2019). Small farms, 1.5–2.0 ha, develop an important role in the whole EU oil production, contributing to food security, improving the living conditions of the peoples in the rural areas, as well as protecting ecosystems. About 400 olive cultivars, spread on the Italian territory, provide the greatest biodiversity in the EU countries (ISMEA 2019). In small farms, olives are collected by hand and stored in ventilated boxes, until the time of processing (Figure 4).

With the availability of many milling plants, both small and large farms can process their olives within 24–48 h, thereby increasing the organoleptic and nutritional quality of the EVOO. A similar quality level is harder to be reached, when only a few large oil mill plants, would be available for many farms in a wide territory.

The olive processing technology is characterized by a strong innovation. The traditional pressure systems, called discontinuous method, is no longer used, because of several applicative problems, linked to lower productive capacity and cleaning difficulties [39]. The modern milling plants comprise both small oil mill extractors, working on 80–100 Kg olives/hour and bigger olive oil plants endowed with conventional three-way decanters (3W) or with two-way decanters (2W), able to process 300 to 600 Kg/h. The decanter is used for separating oil, olive pomace and water at the 3W system; oil and olive pomace at the 2W system, from the olive paste. In a previous paper [10], we showed the relevant differences in the phenol content due to the change of the 3W decanter with the 2W decanter in the milling plant. Two cultivars of olives (*Raggiola* and *Leccino*) were harvested in the same day and then divided for milling. The results showed a marked increase of oleuropein aglycone, oleacein, oleocanthal, as well as of the lignans in the 2W system. The individual phenolic compounds were obtained by the HPLC method and their sum was confirmed by the total phenol assay, made with the Folin–Ciocalteu reagent [10]. For instance, in the case of cv. *Raggiola*, the 2W system provided + 30% total phenols than the 3W system [10]. 

Not only the decanter, which is the central part of the modern olive oil plant, but also the crushing and the malaxation systems have been deeply investigated by many research groups, to find devices for protecting the antioxidant pool of the olives from degradative reactions [34]. Interestingly, the use of inert gases (N2) in the malaxation chamber reduced significantly the losses of antioxidants [34]. A new approach has been developed by the rapid temperature optimization during malaxation, obtained with flash thermal conditioning, which showed significant improvement in terms of volatile compounds and secoiridoids [40]. The traditional malaxation temperatures were first increased to 25–30 °C then followed by cooling to inactivate the lipoxygenases and the polyphenol oxidase [40].

Indeed, enzymes such as pectinase, hemicellulase, and cellulase able to degrade the pectic substances, have been experimented for improving the oil yield and the concentration of polyphenols [41]. Pectinases and cellulases are naturally present in the olive, but they are deactivated during the processing by oxidized phenols, bonding the enzyme prosthetic group [42].

As the phenol concentration allows to make claims, regarding the oil’s health benefits, every improvement of the technology aimed to increase the quality level in terms of antioxidants is pursued. Therefore, the availability of modern extractive processes, combined with the olive harvest in the high-phenol ripening stage and short storage times before milling, become important factors to reach the highest nutritional quality.

## 4. Antioxidants in Conventional and Organic EVOO

The composition of phenolic compounds of the olive fruits strongly depend on the agronomic practices. The most studied practices include fertilization and irrigation. In both cases, results provided clear guidelines. For instance, concerning irrigation, it was shown that there is a negative correlation between the amount of water and concentration of the secoiridoid derivatives in EVOO, whereas the lignans behave contrary wise [14,43,44].

As far as the fertilization is concerned, the increase of the N fertilization depletes the secoiridoids of olive fruit [45,46]; whereas, the increase of the *K* applications, does not affect the polyphenols [47].

Regarding the comparison between organic and conventional agronomic practices, the results are controversial. Therefore, it is not possible, with the available data, to state the superiority of one practice versus the other, in terms of nutrient contents. Some studies were earlier performed along one year and not all variables were carefully controlled. For instance, Gutierrez et al. [48] compared conventional and organic EVOO, in one year, with one olive cultivar at ripening index variable from 3.5 to 5.0. Conventional fertilization made with urea and potassium nitrate was not fully described. The authors showed an increase in tocopherols, polyphenols and oleic acid in organic oil versus the conventional one [48]. Later, we performed a three years’ study to compare EVOOs produced from two olive cultivars, cultivated in two orchards, located in the same village and exposed to the same sunlight and atmospheric precipitation [49]. The olive harvesting time and the maturation degree were the same for each cultivar. In the oils, we detected only differences in taste parameters but the phenol content showed, in the three years, greater differences linked to climate changes than due to different agronomic practices [49]. The analysis was performed on the oil amounts prepared for the market and not on samples prepared with small lab mills [49].

Another research group analyzed both fruit and oil characteristics under organic versus conventional practice in two olive cultivars [50]. Fruit characteristics, including maturation, fruit pulp and, pit weight, were not significantly different either among treatments or within the cultivars. Olive samples were harvested and EVOOs were extracted with a lab mill to prepare the experimental oil samples. No differences were found in merchandise parameters, while polyphenols were strongly reduced in the conventional system [50]. The fatty acids composition was similar among treatments except for a slight reduction of linoleic acid in the conventional EVOO; the sensory evaluation confirmed more bitterness and pungency in the organic EVOO [50].

Recently, the comparison between organic vs. conventional EVOOs was carried out by Lopez-Yerena [51]. The oil extraction was made on one kg of olives with a lab milling reproducing the industrial process. The authors found a high level of secoiridoids (+30%) in the organic than conventional EVOO. On the contrary, the lignans were higher under the conventional system: whereas, the flavonoids, apigenin and lutein, were not affected by the agronomic practices [51]. The authors analyzed also the effect of the ripening index on total polyphenols and individual secoiridoids; they confirmed that, in both agronomic systems, the secoiridoids decreased in the same way with the olive ripening stage [51].

All cited authors agree on the need to set up experiments to eliminate the effect of the seasonality as well as to fix the parameters, which clearly distinguish the organic and the conventional practices. This difficulty lies probably in the fact that the definition of organic and conventional practices is too broad and different techniques are enclosed in the same system [50]. Furthermore, the different location of the olive orchards introduces differences in the olive exposition to light, which is an important determinant for affecting the phenols content [52]. Therefore, a wide number of parameters must be controlled to detect whether the nutritional quality of organic fruits is higher than the conventional ones.

## 5. TO Technology

**Figure 5:**
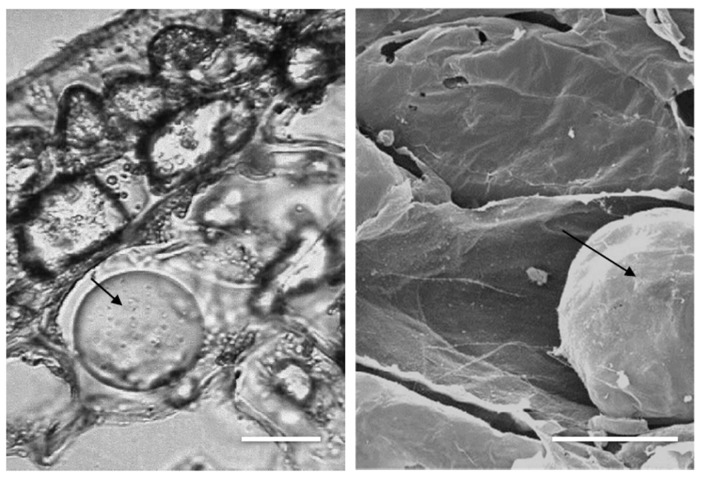
Olive mesocarp cells with oil droplets (arrows) by (**left**) light microscopy and (**right**) scanning electron microscopy. Bars = 30 μm.

Olive mesocarp represents 70–85% of the fruit and together the epicarp constitutes the edible portion of the olive fruit. Generally, the nutritional composition on the nutritional label is related to 100g of edible portion (e.p.). Mesocarp principally contains water, lipids, sugars, acids, pectic substances, cellulose and minor constituents as biophenols, vitamins, pigments and minerals. The oil droplets are present in the cell vacuole and constitute the 10–25% of the fruit weight (Figure 5). Both vacuolar and drop-like inclusions of biophenols were detected in epidermal, hypodermal and mesocarpal cells and show a homogenous distribution from epicarp to lower mesocarp [53].

The table olive (TO) production technology is quite simple and follows traditional methodologies although they are prepared by many processing methods, each one can affect in a different way, the phenol composition and the nutritive value [25,54,55,56,57,58]. The principal treatments are two, with some exceptions: a) chemical debittering of the fruit by lye (treated olives); b) biological debittering of the fruit by microorganisms (natural olives). The “treated olives”, according to IOC “Trade Standard Applying to Table Olives” [59] are “green olives, olives turning color or black olives that have undergone alkaline treatment, then packed in brine in which they undergo complete or partial fermentation, and preserved or not by the addition of acidifying agents”. The exception is the Castelvetrano method, in which the olives are kept directly in an “alkaline brine” (NaOH and NaCl). The “natural olives”, according to IOC “Trade Standard Applying to Table Olives” [59] are “green olives, olives turning color, or black olives placed directly in brine in which they undergo complete or partial fermentation, preserved or not by the addition of acidifying agents”. The exception is the Itrana method in which the olives are immersed in water for 30–40 days before brining, to promote development of specific debittering microorganisms (yeasts and lactic acid bacteria). Both fermentation steps involve a diversified microflora composed of lactic acid bacteria and yeasts. A good colonization of tissues by microorganisms, together the osmotic exchange olive/brine of biophenols and sugars, accelerates the fermentative process. Glycosides oleuropein, demethyloleuropein, ligstroside, and verbascoside are significantly reduced and, also the related aglycons (3,4-DHPEA-EDA and *p*-HPES-EDA).

If the batches are inoculated with oleuropeinolytic bacteria, the increase of simple phenols, such as hydroxytyrosol is evident (Table 1) [60].

All table olives, analyzed by different authors are, despite treatments, still rich in natural antioxidants (at least 30 different compounds) such as polyphenols, vitamins, triterpenic acids, and sterols (Table 2). It was demonstrated that the variability of those compounds can be related to a combination of several factors such as: cultivar, stage of ripening, climate conditions, water regime, agronomic practices and technological processes.

Regarding to vitamin content, table olives of *Intosso d’Abruzzo* cv [54] are rich in tocopherols and tocotrienols. The most abundant is α-tocopherol (vitamin E), with a concentration of 6.44 mg/100 g of e.p. (Table 3). On the basis of the Regulation (EC) N. 1924/2006 [70] on nutrition and health claims made on foods, confirmed by Regulation (UE) N. 432/2012 [28], a claim that a food is a source of vitamins may only be made where the product contains at least a significant amount as defined in the Annex to Directive 90/496/EEC [71] and substituted by Annex of Directive 2008/100/CEE [72]. For vitamin E, the significant amount for 100 g of e.p. is 15% of RDA specified in the Annex (12 mg). These olives provide, also, discrete amounts of A group vitamins, considered to have great antioxidant effects. The ascorbic acid (vitamin C) content is <1 mg/kg for e.p. but, being added as an antioxidant in some preparations, its content increases in the end product.

On the other hand, table olives are a good source of dietary fiber, which also has a high digestibility rate [25,73]. On the basis of the Regulation (EC) N. 1924/2006 [70], it is possible to write on the commercial label the claim “source of fiber” if the product contains at least 3 g of fiber/100 g of e.p. Most preparations have a content of fiber ≥ 3 g/100 g of e.p. [25], so they can be considered as a source of fiber. A recent study [74] shows that the consume of “antioxidant-rich dietary fiber” (ADF), which is achieved by combining dietary fiber and antioxidants such as biophenols, could reduce the incidence of cardiovascular disease. However, some doubt has been raised on the absorption of phenols through the intestinal cells as the digestive process could be slowed by the presence of the fiber, which seems to retain phenolic compounds [75]. On the basis of the few published data on intestinal absorption of TOs phenols, it results that the overall bioavailability of 3,4-DHPEA and p-HPEA was 1.86% [76]. On the other hand, it has been reported that absorption of 3,4-DHPEA and p-HPEA from EVOO was 30–60% and 20–22% respectively, of the total amount and p-HPEA-EDA reached absorption values of 60–90% [77].

## 6. EVOO Antioxidants and the Cooking Methods

The use of EVOO in the coking practices may have positive or negative effects on the nutritional content. The thermal treatment contributes to one side to release nutrients from foods but, on the other side, it oxidizes phytochemicals and fatty acids, thus reducing EVOO health benefits. The best option is to use EVOO on the vegetables after cooking or at the end of the cooking. Few studies on the health benefits of EVOO have distinguished between raw oil and cooking oil. The common domestic EVOO use include: frying, soups and stews in oven. In frying, the polyphenols stability is influenced by the composition of the oil, cooking temperature, time and type of food present. Studies showed about - 60% decrease in secoiridoids after 30 min cooking of the oil alone and about 90% after 60 min of heating [78,79]. Silva et al. [80] showed a less marked depletion of phenols in olive oil used for frying. The oleocanthal was the most stable secoiridoid: the hydroxytyrosol was completely depleted by the heating process; the lignans were relatively heat stable [81]. 

In a previous paper [82], we measured the peroxide number and the polyphenols in EVOO sauteed 15 min alone or in the presence of a vegetable mixture, namely onion, celery, carrot and garlic, used for the Italian soffritto. An EVOO heated 15 min at 180 °C, showed a 45% depletion of polyphenols, whereas the peroxide number increased from 5 to 22 meq O_2_/Kg. In the presence of the vegetable mixture, the polyphenols of the same EVOO decreased about 30% with the peroxide number reaching 10 meq O_2_/Kg. In the same study, data were compared with sunflower oil, where the polyphenols decreased from 20 to 3 mg/Kg in the oil alone and from 20 to 8 mg/Kg in the oil with the vegetables [82]. The peroxide values increased from 1 to 38 meq O_2_/Kg in the oil alone and to 27 meq O_2_/Kg in the presence of the vegetables: the oil acidity was not modified by heating either with the oil alone or in the presence of the vegetables [82]. Results indicated that the EVOO when mixed to the vegetables, maintained most of the phenolic compounds and extracted those of the vegetables by forming an antioxidant mixture able to increase the EVOO stability to heat. The dressing mixture for pasta, completed by the addition to the vegetable mixture of the tomato juice, with its lycopene, provided a huge amount of antioxidants, which makes the dressed pasta dish a healthy food, able the reverse the oxidative stress, which occurs after every meal [82].

## 7. TO Antioxidants, Thermal Treatments and Shelf-Life

Due to new lifestyle and alimentary behavior, TO consumption is growing quickly. The Italian cuisine offers many dishes, starter, appetizer, street food and finger food in which olives are an essential ingredient: table olive-based condiments for pasta, pizza, pies, “bruschetta”, sandwiches, salads, bread-dough mixed with green/black olives. Sometimes, to enhance the flavor of table olive products and increase their resistance to oxidation, spices and aromatic herbs as oregano, wild fennel or rosemary, containing substances as volatile oils, oleoresin, L-ascorbic acid, and biophenols, are added to the products [83]. TOs can be offered on the market in several styles and presentations, which include: “stoned or pitted olives” (olives from which the stone/pit has been removed, without affecting their natural shape), and “olive paste or pate” (finely shredded olive flesh derived by stoned olives). Both of these products could be preserved in EVOO, possibly of the same olive variety. The composition and the oxidative stability of the covering olive oil, as well as the pasteurization treatment, necessary for this type of products, influences the nutritional and sensory properties of end product. In the study of Lanza et al. [84], the Taggiasca EVOO before the treatments was characterized by a fair amount of biophenols (264–245 mg/kg oil) and tocopherols (218–222 mg/kg oil). The content of biophenols and tocopherols decreased after pasteurisation and during storage, and this trend was more relevant in olive paste, probably due to the contact of the oil with a greater surface area of broken olive tissues that accelerated the degradation processes. At the end of storage, the amount of TO biophenols reaches ~100 mg/kg oil and tocopherols reach the value of 5 mg/kg oil. Thermal treatments, such as pasteurization, mostly impact on hydroxytyrosol [85].

## 8. Conclusions

In the future market, the food nutrient level will be increasingly important, because of the demand for high-quality products required by EU citizens (EUROBAROMETER, http://ec.europa.eu/public_opinion/archives/eb/eb80/eb80_en.htm). The EVOO is one of the most important health-protective foods in the Mediterranean diet and the EFSA has admitted the health claim after the secoiridoids certification. Actually, many farmers possess basic agronomic and technological abilities, as a result of information campaigns, made by the institutional organisms to reach the standard quality for the EU health claim. However, this does represent yet neither a quality parameter for consumers nor a resource for increasing the economic income of the farmers. In fact, an official method for the determination of secoiridoidic structures of tyrosol and hydroxytyrosol is still lacking. The discussion inside the IOC panel of analytical expertise regards the opportunity to adopt and validate a method of analysis, which includes the hydrolysis or not [26]. Figure 4 shows in one draft the EVOO food chain and highlights the need for the conclusive analytical step. This picture would be a memorandum for the IOC scientists and a wish to conclude in a short time their work, with an agreement on a validated method.

A second important issue for the EVOO studies is to search for new olive cultivars more tolerant or in the best case resistant to the *Xylella fastidiosa* attack. The results obtained with the olive cultivar FS17 are encouraging, but further research is needed to search for new cultivars and remedies to save the EVOO production [86,87]. The *Xylella fastidiosa* attack represents a true disaster, which was it causing the loss of 4 million of olive trees and 29,000 tons of EVOO in one year in Italy.

Another important issue is the modernization of olive milling plants and the storage tanks. Storage under conditions protected by the light and oxygen, possibly by still tanks with an N_2_ head-space at pressure (0.02 ATM) are necessary.

The cooking practices must be performed with minimum heat stress and studies for demonstrating the best condition to minimize the phenol losses should still be carried out. For instance, the sous vide cooking method must be tested for the EVOO antioxidants maintenance, at low temperatures for long exposure times.

The use of vitaminized EVOO is another important issue to be developed, due to the property of EVOO to be a good solvent for lipophilic vitamins and useful food carrier in dietary supplements [88].

Finally, cosmetology is another important sector where EVOO is used. In fact, antioxidants of EVOO exploit anti-inflammatory activity and in combination with vitamin E, D, and K as well as carotenoids, guarantee nutrition and protection to the skin against UV arrays and dehydration [89,90]. Cosmetology based on EVOO has generated research and development products able to protect irritated skins, when threatened by skin pathologies, like psoriasis and eczema. Many products are actually on the marketplace based on the antioxidant properties of EVOO, but their use will require further improvement for consistent beneficial effectiveness.

Referring to the TO composition (high bio-phenols content with antioxidant and radical scavenging activity, vitamins, MUFA, PUFA, minerals and other nutraceutical compounds), it appears quite clear that TOs already have most of the characteristics required to properly join the group of “functional foods” because they have a potentially positive effect on health beyond basic nutrition. The main purpose of food industries is to formulate new types of food, fortified with probiotic bacteria, that are released only after reaching the human gastrointestinal tract and exerting an equilibrium action on the intestinal microflora, through direct colonization and in quantities sufficient to improve the consumer health. Therefore, table olives, fermented with probiotic strains, preferably isolated from the microbiota, that colonize the surface and the interior of the olive itself, will become a real functional food.

## Figures and Tables

**Table 1:** Effect of spontaneous and inoculated fermentation on phenolic composition of table olives. Data are expressed as mg/kg of pulp dry weigh. From Servili et al. [60].

Phenols		Frantoio			Leccino	
	Raw	Spontaneous	Inoculated	Raw	Spontaneous	Inoculated
Hydroxytyrosol	293	357	490	567	693	1169
Tyrosol	123	121	112	165	163	158
Oleuropein	505	460	nd	1451	1377	230
Demethyloleuropein	1115	991	215	904	803	156
Verbascoside	886	787	663	2297	2040	773
3,4-DHPEA-EDA	2644	2576	1536	5840	5691	2936
*p*-HPEA-EDA	23	21	8	106	103	nd

**Table 2:** Maximum values of antioxidant molecules in table olives (TOs) related to cultivar and technology.

Antioxidant Molecules	Max Value	Cultivar	Technological Process	Reference
Phenolic alcohols				
Hydrotyrosol	2119 **	Coratina	Greek inoculated	[60]
Tyrosol	245 **	Coratina	Greek	[60]
Phenolic acids				
Caffeic acid	318 *	Peranzana	Greek	[61]
*p*-Cumaric acid	10 **	Crete	Greek	[62]
Ferulic acid	3 *	Chetoui	Greek inoculated	[63]
*p*-Hydroxybenzoic acid	10 **	Crete	Greek	[62]
*p*-Hydroxyphenylacetic acid	60 **	Tsakistes	Greek	[62]
3,4-Dihydroxyphenylacetic acid	100 **	Tsakistes	Greek	[62]
Protocatechuic acid	70 **	Crete	Greek	[62]
Syringic acid	4 *	Chetoui	Greek inoculated	[63]
Vanillic acid	26 **	Ascolana tenera	Greek	[64]
Secoiridoids and derivatives				
Oleuropein	3403 *	Nocellara del Belice	Spanish	[65]
Demethyloleuropein	2013 **	Coratina	Greek	[60]
Ligstroside	418 *	Nocellara del Belice	Spanish	[65]
Oleoside 11-methylester	279 *	Nocellara del Belice	Spanish	[65]
3,4-DHPEA-EDA	8987 **	Coratina	Greek	[60]
*p*-HPEA-EDA	103 **	Leccino	Greek	[60]
Hydroxycinnamic acid derivatives				
Verbascoside	2093 **	Coratina	Greek	[60]
Isoverbascoside	4164 *	Peranzana	Greek	[61]
Lignans				
1-Acetoxypinoresinol	39 *	Nocellara del Belice	Spanish	[65]
Pinoresinol	2 **	Itrana	Greek inoculated	[66]
Flavonoids				
Luteolin	801 **	Mele	Lime and ash	[55]
Luteolin-7-*O*-glucoside (cynaroside)	30 *	Nocellara del Belice	Castelvetrano	[65]
Apigenin	23 **	Tonda di Cagliari	Greek	[67]
Apigenin-7-*O*-glucoside	3 **	Verdeal Trasmontana	Alcaparra stoned	[68]
Quercetin-3-rutinoside (rutin)	46 **	Cellina di Nardò	Greek inoculated	[69]
Cyanidin-3-rutinoside	35 **	Cellina di Nardò	Greek inoculated	[69]
Tocopherols				
α-Tocopherol	59 **	Conservolea	Greek inoculated	[69]
Triterpenic acids				
Maslinic acid	2133 **	Conservolea	Greek inoculated	[69]
Oleanolic acid	1333 **	Kalamata	Greek inoculated	[69]
Phytosterols				
Total sterols	1863 ***	Majatica	Oven-dried	[56]

* mg/kg of pulp DW; ** mg/kg of pulp FW; *** mg/kg of oil fraction.

**Table 3:** Vitamin pattern of *Intosso d’Abruzzo* cv. table olives. From Lanza et al. [54].

Vitamin	Quantity
Vitamin C (mg/kg)	<1
Provitamin A carotenoids	
β-criptoxantin (mg/kg)	<0.1
13-*cis*- β-carotene (mg/kg)	0.1
All-*trans*- α-carotene (mg/kg)	<0.1
All-*trans*- β-carotene (mg/kg)	0.7
9-*cis*- β-carotene (mg/kg)	<0.1
Retinol equivalents (μg/100g)	12
Vitamin E (mg/100g)	6.44
